# Latina Women in Academia: Challenges and Opportunities

**DOI:** 10.3389/fpubh.2022.876161

**Published:** 2022-04-26

**Authors:** Ana F. Abraído-Lanza, Sandra E. Echeverria, Karen R. Flórez, Sonia Mendoza-Grey

**Affiliations:** ^1^Department of Social and Behavioral Sciences, School of Global Public Health, New York University, New York, NY, United States; ^2^Department of Public Health Education, School of Health and Human Sciences, The University of North Carolina at Greensboro, Greensboro, NC, United States; ^3^Department of Environmental, Occupational and Geospatial Sciences, Graduate School of Public Health and Health Policy, The City University of New York, New York, NY, United States; ^4^Department of Sociomedical Sciences, Mailman School of Public Health, Columbia University, New York, NY, United States

**Keywords:** Latinas in higher education, public health, racism, academia, oppressed group

## Abstract

Latina women and other ethnic and racial groups continue to be underrepresented in science, technology, engineering, and mathematics (STEM) fields, including public health. This underrepresentation of people from diverse backgrounds and lived experiences in academic public health and other scientific disciplines is a form of epistemic oppression, exclusion that hinders contribution to knowledge production and advancement. Our analysis of 2021 data from the Association of Schools and Programs of Public Health indicates that Latinos/as represented only 6.0% of all instructional faculty and 6.1% of all tenured faculty at schools and programs of public health. We discuss the ways in which sociopolitical contexts, family-level dynamics and gendered norms, and institutional contexts hamper Latinas' full participation in academia. We propose solutions such as redefining metrics for success, leadership accountability, equity analyses, cluster hiring initiatives, and instituting structured mentoring and leadership programs. Bold actions are needed if we are to advance the scientific enterprise and address the diversity and equity problem in public health.

## Introduction

Despite the goal of the field of public health to promote the health of the public and to address health disparities and its commitment to social justice, certain ethnic and racial groups continue to be under-represented (UR) among the faculty of schools and programs of public health (1–3). For these reasons, Ramirez-Valles (4) asserted that the field of public health continues to struggle with an equity problem. Moreover, the under-representation of people from diverse backgrounds and lived experiences in academic public health and other scientific disciplines is a form of epistemic oppression, defined as systematic exclusion that hinders contribution to knowledge production and advancement (5). We concur with scholars who cogently argue that epistemic oppression limits and creates inherent flaws in scientific research (6). Further, the intersection of gender and ethnicity create additional barriers, such that women of color are particularly under-represented especially in academic and administrative senior positions, and face multiple challenges in academic career progression (2, 7, 8).

Several excellent reviews revealed the critical gaps that remain in advancing women in the sciences, including public health (1, 2, 9–11). This perspective article is not based on an extensive review of relevant literature on this topic. Instead, we target key articles for discussion and build on the existing body of work. In addition, we write this perspective from our own lived experience as first generation scholars (offspring of parents who did not earn a college degree), first or second generation immigrants, and as the first or only Latina women in our respective departments and schools, at one point or another in our careers. By centering this review on our lived experience, we hope to highlight how the struggles women faculty of color often experience are rooted in structural, systemic conditions.

We begin by providing a brief overview of trends on Latinas in academia, discuss the ways in which racism and a variety of other structural determinants limit the representation of Latinas and other groups in academia, and propose solutions to address the diversity problem in academic public health by highlighting the importance of resources, initiatives and mentoring that make a difference for Latinas and other women of color in the academy to be seen, affirm their voice, and advance their careers (3, 4, 12).

## National Trends on Latina Representation in Academia

Data from the National Center for Education statistics demonstrate the significant shortage of Latinas in higher education. Among full-time instructional faculty employed in degree-granting postsecondary institutions in 2019 (most recent data available) women represented 47.2% of all faculty (*n* = 398,165), increasing from 31.8% in 1991 (*n* = 165,213). In contrast, in 1991, Latinas represented <1% of all full-time faculty (0.78%, *n* = 4,069) rising only to 2.6% (*n* = 21,814) in 2019.

A study by Goodman et al. (1) demonstrates that these striking disparities are also evident in academic public health. Specially, the authors found that from 1997 to 2017, individuals of Latino/Hispanic origin increased by 2% at schools of public health and represented 8.5% of all primary instructional faculty at schools and programs of public health.

For the present article, we retrieved 2021 data from the Association of Schools and Programs of Public Health online data center, and found that Latinos/as (*n* = 330) represented only 6.0% of all instructional faculty (*n* = 5,485) and 6.1% (*n* = 149) of all tenured faculty (*n* = 2,453) at schools and programs of public health in the United States (49 of these are at the University of Puerto Rico Graduate School of Public Health). Although these percentages are small, tenured Latinos constitute a larger proportion than Black faculty, who only represent 4.3% of all tenured faculty. Underrepresentation of Black faculty merits a thoughtful critical discussion that is beyond the scope of this perspective piece (13). We also found a huge gender gap across ranks when comparing Latino/a men and women. At the Assistant Professor level, Latinas (*n* = 82) far outnumber Latino men (*n* = 36), representing 69.5% of Hispanic faculty at this rank. This advantage continues at the Associate Professor level, with 62.3% of Latinas holding this rank compared to 37.7% of Latino men. However, at the full Professor rank, this trend inverts between Latina women and men (44.3% vs. 55.7%, respectively).

## Structural Contexts

In the following sections, we focus on sociopolitical contexts, family-level dynamics and gendered norms, and institutional contexts that hamper Latinas' full participation in academia.

### Sociopolitical Context

The immigration climate of the nation has a profound effect on Latino scholarship. In recent years, we have seen a retrenchment of inclusive immigration policies and increased criminalization of immigrants (14). From 2008 to 2013, the Secure Communities program grew from 14 jurisdictions in the US to all 50 states (plus the District of Columbia) and allowed the sharing of law enforcement information with federal immigration enforcement authorities. During the 2000's, annual immigration to the US fell by almost half to about 600,000 people per year, a level not seen since the 1980's (15). Some of the authors of this manuscript have direct experiences with parents or close family members who have been undocumented or continue to live through this reality. Indeed, these experiences have been the impetus behind some of our careers, but they also exert personal tolls with practical consequences for advancing our research. Recruiting Latino/a participants for research studies, for example, under this political climate requires a substantial investment of time, trust building, and connecting with organizations that can serve as brokers and allies for our research. This additional level of investment is often not borne by our non-Latino colleagues. Moreover, current US immigration policy has been crafted to source the labor needs of the American economy producing a highly bifurcated system of geography and education, at times favoring some groups while dehumanizing others (16). Thus, the social capital needed to maneuver through the appointment, tenure and promotion process can uncover and deal with traumatic processes of oppression and marginalization that our families and communities have experienced. Examples of programs designed to build social capital include (among others) New Connections and Health Equity Scholars for Action, which link new Latina and other underrepresented scholars and investigators with established researchers, who themselves may have successfully dealt with issues of oppression (17). Lastly, while we are heartened to see that some universities have put policies in place for faculty to document research challenges related to the COVID-19 pandemic, it is not clear if similar processes have been instituted to account for the racial reckoning the nation has recently undergone, including the very visible anti-Black and anti-immigrant policies and practices that shape American life.

### Family-Level Dynamics and Gendered Norms

Families and caregiving responsibilities are not inherent barriers to success in academia. However, they produce differential outcomes across gender when norms and expectations by those in academic institutions stem from the assumption that all faculty have substantial spousal support in the form of a stay-at-home partner (18). Stay-at-home support in the form of a spouse has historically been perceived to be an advantage in academia since it allows for intensive work schedules that maximize the kind of scholarly output that is most revered (e.g., publications, grants). However, about 90% of the spouses of women in academia work outside the home full time, in contrast to 50% of their male counterparts (18). Single women in the academy may be able to adhere to this intense work schedule at first glance, but they also report having less work balance than their married colleagues (19). Although Latinas report more work balance than Latinos (19), there is a dearth of research documenting Latinas caregiving responsibilities such as aging parents or disabled family members. Moreover, regardless of marital status, women in academia who wish to have their own biological children must contend with the inherent conflict between building their careers in the early stages at the same time in which they may need to make crucial family and reproductive decisions (20). Further, Latina faculty also cite the added burden caused by the “push and pull” of their roles as exemplars and mentors to students of color while simultaneously feeling the pressures of their roles as mothers and spouses (21). A study of highly-ranked science programs revealed that female faculty have fewer children than their male colleagues, and nearly twice as many female faculty as men reported having fewer children than they desired because they pursued a science career (22). Career-building and family tensions may be particularly problematic for women pursuing academic careers in global public health, given the need to spend considerable time abroad (7). These conflicts are exacerbated whether or not parental roles are achieved biologically given the lack of integration between family and work in the academy. Indeed, work/family balance is commonly cited as a barrier to academic success among faculty of color (23), and family pressure is still one of the top reasons cited by women exiting tenure track positions in academia (18).

### Institutional Contexts

#### Discrimination and Bias

Structural contexts also include institutionalized racism, bias and discriminatory practices. In their review of gender and ethnicity in 15 leading social science and public health universities, Khan et al. (2) identified broad structural factors that create barriers to diversity in schools of public health. Importantly, they found that gender and ethnicity interact, creating obstacles especially for women of color as they attempt to climb academic ranks. Their analysis revealed that the proportion of ethnic minority women declined from mid- to senior-level academic ranks in all 15 of the universities that they examined. They concluded that marginalization, prejudice and discrimination against ethnic minority women account for these findings. These are manifested, for example, in lower pay for similar positions, temporary contracts, and other practices that lead to lower chances of recruitment and promotion for ethnic minority women.

Other structural factors are at play affecting women's scientific pursuits. For example, there is a lack of representation of women on NIH review panels, and funding gaps. Shen (24) reported that over a period of approximately a decade, a Freedom of Information Act request from *Nature* revealed that the percentage of women on NIH review panels barely shifted from 25% in 2003 to 30% in 2012. Interestingly, these figures mirror approximately the percentage of women applying for and receiving grants during that period. There are also funding gaps, such that in 2012, the NIH awarded 30,768 to men and 13,025 grants to women. Moreover, the average amount of the award was higher for men ($507,279) than for women ($421,385) (24).

There is also evidence that different standards are applied to scholarship dealing with diversity-related topics. For example, one analysis revealed bias against manuscripts dealing with diversity topics such that in early rounds of the review process, diversity manuscripts relative to non-diversity papers were 12 times more likely to be rejected than accepted (25). Moreover, using an experimental design, editorial board members who were asked to review abstracts of manuscripts showed evidence of applying “stricter standards” when evaluating diversity papers, such that quality of manuscripts was associated with editorial decisions for diversity papers but not for other topics (25). Given the importance of publications in obtaining academic positions and achieving tenure and promotion, Latinas and other scholars engaged in social justice and diversity-related research may be subject to biased review of their work, putting their academic opportunities and career progression at risk.

#### Diversity Climate

A “critical mass” of groups that have been historically underrepresented in academia can support a climate of diversity and inclusion. The relatively small numbers of African American, Latino/a, and Native American students and faculty in institutions of higher education may contribute to the perception that they do not “belong.” Several studies document the importance of sense of belonging in academic settings (11, 26, 27). Because of their profound social significance, race or ethnicity contribute to self-efficacy, learning experiences, and choices. Due to racism and other psychosocial processes in the social environment, race/ethnicity is differentially related to a variety of “opportunity structures,” including exposure to role models and mentors that can facilitate (or limit) skills and outcome beliefs [(28), p. 103].

A sense of belonging and self-efficacy also must be studied in the contexts of successes and failures. Many programs encouraging academic achievement among UR groups tend to focus on the pathways and processes of success. Rarely do they discuss the recovery from failures or setbacks. Yet, in academic settings, achievement often is met with setbacks along the path to success. Some examples of setbacks include rejection of a scientific manuscript submitted for publication, an unsuccessful attempt at promotion, a mediocre or unsatisfactory annual performance review, and a poorly-scored grant application. In academic institutions with few students and faculty of color, setbacks or failures may be magnified for UR groups. These magnification effects occur both within UR individuals (i.e., how they perceive themselves) and how others may perceive them. Moreover, given the competitive nature of academia, Latinas and other UR faculty members may not receive guidance and strategies for overcoming these setbacks. Because of their greater presence on campus, those in majority groups who experience a failure may not perceive the event as indication that they do not belong. Similarly, the tendency to value what is familiar may lead to unconscious or conscious “cloning” practices, defined as the tendency of faculty to almost always hire “a clone” of themselves, by powerful institutional committees that make hiring and promotion decisions (29). For example, search committee members may discount or devalue the work or educational credentials of UR job candidates whose academic background and research experiences may differ from their own. “Cloning” can also operate among review, promotion, and tenure committees that are charged with evaluating the performance and career trajectories of UR faculty.

#### Inequitable Academic Practices and Norms

Latinas and other UR faculty members may also be subjected to inequitable service obligations and value conflicts. “Cultural taxation,” the expectation that UR faculty members carry out a variety of diversity service and teaching activities, presents a potential impediment to success (30). Faculty of color often have to teach diversity-related courses, participate in faculty recruitment activities, donate their time and effort to diversity-related training, give guest lectures on diversity-related topics, represent their programs or departments at meetings requiring diversity-related input, serve on search committees requiring minority representation, and so on. Additionally, UR faculty often are made to feel that declining to take on these tasks would undermine the diversity efforts of the University (31). Thus, cultural taxation could adversely affect effort spent on the metrics for success that are more valuable in academic advancement, such as publications and grant-funded research (30).

Moreover, Latinas may experience value conflicts with fundamental norms in academic settings. Self-promotion is central to recognition and advancement in many universities. Members of cultural groups who look down upon self-aggrandizement, however, find it difficult to engage in what is perceived to be “obnoxious self-promotion” activities that are essential to highlight their research, scholarship, and other academic accomplishments (29). UR faculty members also may experience challenges carrying out other normative academic behaviors, such as networking in an unwelcoming environment. Furthermore, UR faculty may not have had similar socialization experiences during their training (access to mentors or extensive networks) that lead to successful integration in the academic profession (31).

## Discussion and Solutions

We end this article by proposing solutions that address the systemic, structural reforms needed to advance the career paths of Latinas and other underrepresented scholars.

*Leadership Accountability*. All of the factors described above present challenges for recruitment, retention, and advancement of Latinas and other UR groups in public health. Institutions cannot address diversity if they do not acknowledge the problem. It is incumbent on the leaders of organizations and institutions of higher education to invest in diversity. Investment includes both financial and social resources. Schools and programs also should be held accountable for analyzing the diversity of their faculty both in hiring, retention, and promotion. There is a need for bold policies that are supported, monitored and enforced by institutional leaders.

*Redefine Metrics of Success*. It is critically important to specify outcomes and indices of how “success” will be defined and measured. This is especially important for UR faculty who engage in diversity-related and community-engaged research. Moreover, institutions should establish systems for tracking retention and advancement. We agree with other scholars who have recommended that institutions should publicly report gender and ethnicity of faculty, including at different seniority levels, and that these data be used for rankings and accreditation [e.g., (2, 4, 9)].

*Pay and Service Equity Analyses*. Institutions, schools and programs should also engage in periodic pay and equity analyses. Tracking of service obligations should include consideration of “invisible” service, such as informal mentoring to students of color to whom the faculty member is not assigned as the advisor of record, or for additional labor provided to students of color in the form of counseling, training and referrals for academic support.

*Cluster-hiring*, in which three or more individuals are hired simultaneously, is another strategy to recruit and retain faculty of color. Cluster hiring eliminates the risk of isolation in departments where the faculty member of color is the “only one.” These circumstances are known to breed feelings of isolation–both social and intellectual–which can lead to professional stagnation or departure from academia (23). Indeed, some programs (e.g., NIH's FIRST) have been launched specifically for the purpose of creating cohorts and communities of underrepresented scientists who are committed to diversity and inclusive excellence. Furthermore, institutions should develop and maintain pipeline programs that include graduate students and postdocs who could transition into early career academic positions with appropriate support.

*Structured mentoring programs and leadership programs* for women can provide needed resources and guidance to UR faculty members. UR mentors who have strategically overcome some of the institutional and other barriers described in the sections above may be particularly well-suited to advise UR students and junior faculty on methods for dealing with these circumstances (32). Race/ethnic-concordant faculty mentors might also serve as important role models that promote UR students' and junior faculty members' perceptions that they, too, can succeed in academic settings. Of course, the lack of established UR researchers who can serve as mentors can become a self-perpetuating cycle (23), underscoring the need to assure that institutional policies, practices and structures contribute to the success of Latinas and other groups in academic settings. Lastly, private foundations and the National Institutes of Health [e.g., (33, 34)] have developed programs to support faculty of color and these programs should be adopted by institutions and offered to faculty, especially early stage scholars.

Taken together, our findings and existing research on women of color in public health highlight the need to end epistemic oppression for all groups, including Latinas. We are encouraged by the growing recognition that structural racism, which includes anti-immigrant policies, permeates all of the work we do in public health. Bold actions are needed if we are to advance the scientific enterprise and address the diversity and equity problem in public health.

## Data Availability Statement

The data analyzed in this study is subject to the following licenses/restrictions: Available only *via* the Association of Schools and Programs of Public Health. Requests to access these datasets should be directed to data@aspph.org.

## Author Contributions

AA-L, SE, and KF contributed to the writing of the manuscript and ideas for sections of the manuscript. SE and AA-L conducted the analyses. SM-G reviewed the existing literature, the findings, and contributed significantly to ideas for the manuscript. All authors contributed to the article and approved the submitted version.

## Funding

This work was supported by the CUNY SPH Health Equity Grant (Grant Number 95790-001) and the Robert Wood Johnson Foundation (Grant Number 77948).

## Conflict of Interest

The authors declare that the research was conducted in the absence of any commercial or financial relationships that could be construed as a potential conflict of interest.

## Publisher's Note

All claims expressed in this article are solely those of the authors and do not necessarily represent those of their affiliated organizations, or those of the publisher, the editors and the reviewers. Any product that may be evaluated in this article, or claim that may be made by its manufacturer, is not guaranteed or endorsed by the publisher.

## References

[1] Goodman MS Bather JR Healton CG Plepys CM Kelliher RM. Racial/ethnic diversity in academic public health: 20-year update. Public Health Rep. (2020) 135:74–81. 10.1177/0033354919887747 31747339 PMC7119260

[2] Khan MS Lakha F Tan MMJ Singh SR Quek RYC Han E. More talk than action: gender and ethnic diversity in leading public health universities. Lancet. (2019) 393:594–600. 10.1016/S0140-6736(18)32609-6 30739695

[3] Rubin R. Despite policies to improve faculty diversity, disparities persist at public health schools. JAMA. (2019) 321:2268–70. 10.1001/jama.2019.3891 31141107

[4] Ramirez-Valles J. Public health has an equity problem: a latinx's voice. Front Public Health. (2020) 8:559352. 10.3389/fpubh.2020.559352 33042954 PMC7517338

[5] Dotson K. A cautionary tale: on limiting epistemic oppression. Frontiers. (2012) 33:24–47. 10.5250/fronjwomestud.33.1.0024

[6] Buchanan NT Perez M Prinstein MJ Thurston IB. Upending racism in psychological science: Strategies to change how science is conducted, reported, reviewed, and disseminated. Am Psychol. (2021) 76:1097–112. 10.1037/amp0000905 34990171

[7] Downs JA Reif LK Hokororo A Fitzgerald DW. Increasing women in leadership in global health. Acad Med. (2014) 89:1103–7. 10.1097/ACM.0000000000000369 24918761 PMC4167801

[8] Talib Z Burke KS Barry M. Women leaders in global health. Lancet Glob Health. (2017) 5:e565–6. 10.1016/S2214-109X(17)30182-1 28495255

[9] Alang S Hardeman R Karbeah J Akosionu O McGuire C Abdi H. White Supremacy and the Core Functions of Public Health. Am J Public Health. (2021) 111:815–9. 10.2105/AJPH.2020.306137 33826395 PMC8033999

[10] Bowleg L. “The master's tools will never dismantle the master's house”: ten critical lessons for black and other health equity researchers of color. Health Educ Behav. (2021) 48:237–49. 10.1177/10901981211007402 34080476

[11] Brannon TN Lin A. “Pride and prejudice” pathways to belonging: Implications for inclusive diversity practices within mainstream institutions. Am Psychol. (2021) 76:488–501. 10.1037/amp0000643 32772541

[12] Moore H. Passing on faculty roles, cui bono? Contexts. (2012) 11:76–9. 10.1177/1536504212466346

[13] Gregory ST. Black faculty women in the academy: history, status, and future. J Negro Educ. (2001) 70:124–38. 10.2307/3211205

[14] De Trinidad Young M Wallace SP. Included, but deportable: a new public health approach to policies that criminalize and integrate immigrants. Am J Public Health. (2019) 109:1171–6. 10.2105/AJPH.2019.305171 31318585 PMC6687277

[15] Frey WH. The 2010s Saw the Lowest Population Growth in U.S. History, New Census Estimates Show. Brookings. (2020). Available online at: https://www.brookings.edu/blog/the-avenue/2020/12/22/the-2010s-saw-the-lowest-population-growth-in-u-s-history-new-census-estimates-show/ (accessed April 7, 2022).

[16] Portes A. Bifurcated immigration and the end of compassion. Ethn Racial Stud. (2020) 43:1, 2–17. 10.1080/01419870.2019.1667515

[17] Health Equity Scholars for Action RWJF. Available online at: https://www.rwjf.org/en/library/funding-opportunities/2021/health-equity-scholars-for-action.html (accessed June 16, 2021).

[18] National Academy of Sciences, National Academy of Engineering, and Institute of Medicine. Beyond Bias and Barriers: Fulfilling the Potential of Women in Academic Science and Engineering. Washington, DC: The National Academies Press (2007). 20669424

[19] Denson N Szelényi K Bresonis K. Correlates of work-life balance for faculty across racial/ethnic groups. Res High Educ. (2018) 59:226–47. 10.1007/s11162-017-9464-0

[20] Powell K. The Parenting Penalties Faced by Scientist Mothers. Nature. (2020). Available online at: https://www.nature.com/articles/d41586-021-01993-x (accessed April 7, 2022).

[21] Saldana LP Castro-Villarreal F Sosa E. “Testimonios” of Latina junior faculty: bridging academia, family, and community lives in the academy. Educ Found. (2013) 27:31–48.

[22] Ecklund EH Lincoln AE. Scientists want more children. PLoS ONE. (2011) 6:e22590. 10.1371/journal.pone.0022590 21850232 PMC3151251

[23] Kameny RR DeRosier ME Taylor LC McMillen JS Knowles MM Pifer K. Barriers to career success for minority researchers in the behavioral sciences. J Career Dev. (2014) 41:43–61. 10.1177/0894845312472254

[24] Shen H. Inequality quantified: mind the gender gap. Nature. (2013) 495:22–4. 10.1038/495022a 23467149

[25] King EB Avery DR Hebl MR Cortina JM. Systematic subjectivity: how subtle biases infect the scholarship review process. J Manage. (2018) 44:843–53. 10.1177/0149206317743553

[26] Hurtado S Ponjuan L. Latino educational outcomes and the campus climate. J Hispanic High Educ. (2005) 4:235–51. 10.1177/1538192705276548

[27] Maestas R Vaquera GS Zehr LM. Factors impacting sense of belonging at a Hispanic-serving institution. J Hispanic High Educ. (2007) 6:237–56. 10.1177/1538192707302801

[28] Lent RW Brown SD Hackett G. Toward a unifying social cognitive theory of career and academic interest, choice, and performance. J Vocat Behav. (1994) 45:79–122. 10.1006/jvbe.1994.1027

[29] Moody J. Faculty Diversity: Problems and Solutions. New York, NY: Routledge Falmer (2004).

[30] Padilla A. Ethnic minority scholars, research, and mentoring: current and future issues. Educ Res. (1994) 23:24–7. 10.2307/1176259

[31] Bensimon EM Ward K Sanders K. The Department Chair's Role in Developing New Faculty into Teachers and Scholars. Bolton, MA: Anker Publishing Company (2002).

[32] Davidson MN Foster-Johnson L. Mentoring in the preparation of graduate researchers of color. Rev Educ Res. (2001) 71:549–74. 10.3102/00346543071004549

[33] Collins FS Adams AB Aklin C Archer TK Bernard MA Boone E. Affirming NIH's commitment to addressing structural racism in the biomedical research enterprise. Cell. (2021) 184:3075–9. 10.1016/j.cell.2021.05.014 34115967

[34] Valantine HA Lund PK Gammie AE. From the NIH: a systems approach to increasing the diversity of the biomedical research workforce. CBE Life Sci Educ. (2016) 15:fe4. 10.1187/cbe.16-03-0138 27587850 PMC5008902

